# Effects of lifestyle intervention on adults with metabolic associated fatty liver disease: A systematic review and meta-analysis

**DOI:** 10.3389/fendo.2023.1081096

**Published:** 2023-02-16

**Authors:** Xiao-Ni Chai, Bing-Qian Zhou, Ni Ning, Ting Pan, Fan Xu, Si-Han He, Ni-Ni Chen, Mei Sun

**Affiliations:** ^1^ Xiangya Nursing School, Central South University, Changsha, China; ^2^ School of Nursing, Hunan Traditional Chinese Medical College, Zhuzhou, China; ^3^ Department of Health Management, The Third Xiangya Hospital, Central South University, Changsha, China; ^4^ School of Nursing, Changsha Medical University, Changsha, China

**Keywords:** MAFLD, diet, exercise, lifestyle, systematic review, meta-analysis

## Abstract

**Introduction:**

This systematic review and meta-analysis evaluates the overall effects of lifestyle interventions upon hepatic fat content and metabolism-related indicators among adults with metabolic associated fatty liver disease.

**Methods:**

It was registered under PROSPERO (CRD42021251527). We searched PubMed, EMBASE, MEDLINE, Cochrane, CINAHL, Scopus, CNKI, Wan-fang, VIP, and CBM from the inception of each database to May 2021 for RCT studies of lifestyle interventions on hepatic fat content and metabolism-related indicators. We used Review Manager 5.3 for meta-analysis and used text and detailed tabular summaries when heterogeneity existed.

**Results:**

Thirty-four RCT studies with 2652 participants were included. All participants were obesity, 8% of whom also had diabetes, and none was lean or normal weight. Through subgroup analysis, we found low carbohydrate diet, aerobic training and resistance training significantly improved the level of HFC, TG, HDL, HbA1c, and HOMA-IR. Moreover, low carbohydrate diet is more effective in improving HFC than low fat diet and resistance training is better than aerobic training in reduction in HFC and TG (SMD, -0.25, 95% CI, -0.45 to -0.06; SMD, 0.24, 95% CI, 0.03 to 0.44, respectively).

**Discussion:**

Overall, this is the first review that systematically synthesizes studies focused on the effects of various lifestyle on adults with MAFLD. The data generated in this systematic review were more applicable to obesity MAFLD rather than lean or normal weight MAFLD.

**Systematic Review Registration:**

https://www.crd.york.ac.uk/prospero/, identifier (CRD42021251527).

## 1 Introduction

Metabolic associated fatty liver disease (MAFLD) is a chronic liver disease characterized by the excessive accumulation of fat in liver cells and metabolic dysfunction (1, 2). MAFLD, formerly known as non-alcoholic fatty liver disease (NAFLD), is redefined in the guidelines of the Asia-Pacific Society of Liver Diseases in October 2020. The new definition of MAFLD is based on the presence of fatty liver as indicated by liver biopsy or imaging or blood biomarkers, along with one of three conditions: overweight/obesity, type 2 diabetes, and metabolic dysfunction. New definition attaches importance to the pathogenesis of MAFLD and makes change to end points of study, which differ from NAFLD that excludes alcohol consumption (3). MAFLD affects approximately one quarter of the global population, which not only causes liver inflammation, fibrosis, and malignant tumors, but also often merges with a variety of metabolic disorders, causing major diseases such as gout, type 2 diabetes, hypertension, and atherosclerosis and posing a major health and economic burden to all societies (1, 4–6). Therefore, an effective approach to address such a serious situation is urgently needed.

The high prevalence of MAFLD is fueled by the rapid rise in unhealthy lifestyle including sedentary behavior and unreasonable dietary structure (7). Currently, lifestyle interventions are the primary recommended therapy for MAFLD, especially in the absence of approved pharmaceutical agents (1, 8). However, lifestyle interventions for MAFLD patients vary widely among studies, which focus on diet and/or exercise, mainly encompassing Mediterranean diet, Dietary Approaches to Stop Hypertension, high-dietary-fiber diet, aerobic exercise, and resistance exercise (9–15). In addition, the effects of lifestyle interventions differ in the available studies. The effects regarding lifestyle interventions on MAFLD are reflected in the hepatic fat content (HFC) and metabolism-related indicators, such as body mass index (BMI), waist circumference (WC), blood pressure (BP), plasma triglycerides (TG), plasma high-density lipoprotein cholesterol (HDL), fasting blood glucose (FBG), HbA1c, homeostasis model assessment of insulin resistance score (HOMA-IR), and plasma high-sensitivity C-reactive protein (CRP) level, yet these indicators are not fully included in the respective studies (10, 12, 16–18).

Although reviews focus on NAFLD(19–23), to date, none have applied systematic approaches to examine the efficacy or effectiveness of various lifestyle interventions on MAFLD after the name changed and the new diagnostic criteria were redefined. Therefore, we conducted a systematic review and meta-analysis to integrate and evaluate the relevant evidence of various lifestyle interventions in adults with MAFLD and to provide reference for clinical care teams.

## 2 Methods

This study protocol was registered on PROSPERO (CRD42021251527). The study was reported in accordance with the Preferred Reporting Items for Systematic Reviews and Meta-Analyses (PRISMA) guidelines (24).

### 2.1 Eligibility criteria for this study

#### 2.1.1 Types of participants

NAFLD adults who met the diagnostic criteria of MAFLD and adults diagnosed with MAFLD were included in this review. The diagnostic criteria for MAFLD were based on histological, imaging, or blood biomarker evidence of liver fat accumulation, combined with one of the following three conditions: overweight/obesity, type 2 diabetes, or lean/normal weight but presence of at least two metabolic risk abnormalities (2).

#### 2.1.2 Types of interventions

Interventions included lifestyle interventions, such as diet, physical activity, sleeping, or a combination of two or three. Studies designed to prove the effectiveness of dietary supplements or herbal preparations were excluded.

#### 2.1.3 Types of comparators

Comparators included no intervention, standard/usual care, or other lifestyle interventions.

#### 2.1.4 Types of outcomes

The primary outcomes of interest were changes in HFC assessed by histological, imaging, or blood biomarkers. The secondary outcomes included BMI, WC, BP, plasma TG, plasma HDL, FBG, HbA1c, HOMA-IR, and plasma high-sensitivity CRP level.

#### 2.1.5 Types of study

Only randomized clinical trials were included in this review.

### 2.2 Search strategy and study selection

**Figure 1:**
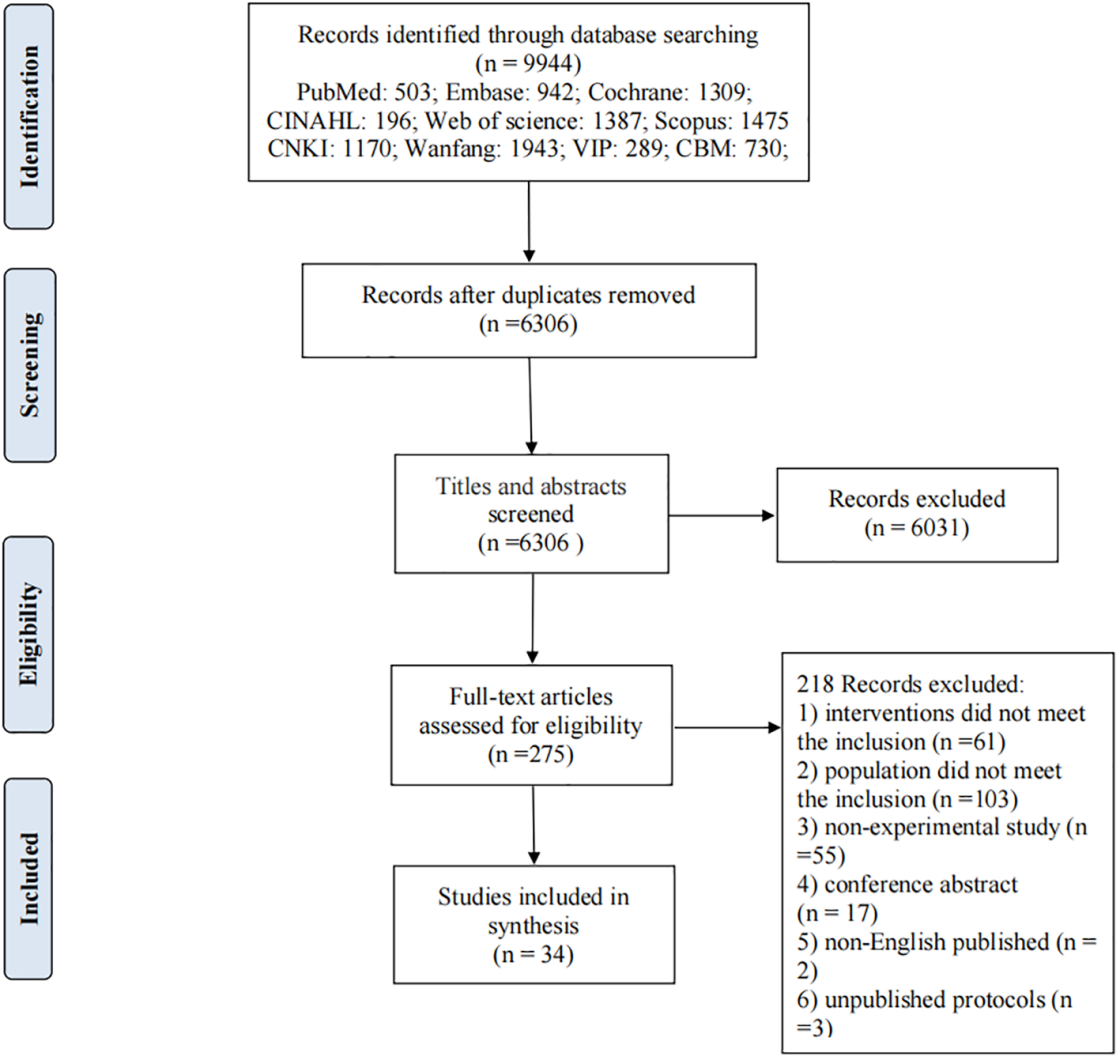
PRISMA flow diagram.

Searches were performed in English and Chinese databases, such as PubMed, EMBASE, MEDLINE, Cochrane, CINAHL and Scopus for English literature and CNKI, Wanfang, VIP, and CBM for Chinese literature. The search period was from the inception of each database to May 2021. The Medical Subject Headings and related free words were widely used to capture the literature, including non-alcoholic fatty liver disease, metabolic associated fatty liver disease, lifestyle, diet, exercise, sleep, and all synonyms of these keywords. The detailed search strategy of PubMed is shown in **Table 1**. Other database search strategies are listed in the Appendix. Articles that satisfied the eligibility criteria and studies published in English or Chinese were included. The screening process included four steps and is shown in **Figure 1**. First, all titles and abstracts retrieved were downloaded to the Endnote X9 library, and duplicate articles were removed. Second, articles were excluded based on titles and abstracts. Third, the full text of the article was reviewed to determine eligibility based on the inclusion criteria. Finally, the reference lists of the included articles and related reviews were screened for potentially relevant articles. The searching and screening processes were conducted by two independent researchers, and disagreements were resolved by a third researcher.

**Table 1:** Search strategy of PubMed.

Main Concept	Search Formula	Results
1. MAFLD	Non-alcoholic Fatty Liver Disease[Mesh] OR Metabolic associated fatty liver disease OR MAFLD OR Metabolic-dysfunction-associated fatty liver disease OR Non alcoholic Fatty Liver Disease OR NAFLD OR Nonalcoholic Fatty Liver Disease OR Fatty Liver, Nonalcoholic OR Fatty Livers, Nonalcoholic OR Liver, Nonalcoholic Fatty OR Livers, Nonalcoholic Fatty OR Nonalcoholic Fatty Liver OR Nonalcoholic Fatty Livers OR Nonalcoholic Steatohepatitis OR Nonalcoholic Steatohepatitides OR Steatohepatitides, Nonalcoholic OR Steatohepatitis, Nonalcoholic	34802
2. Lifestyle	Healthy Lifestyle[Mesh] OR Life Style[Mesh] OR Sedentary Behavior[Mesh] OR Life Styles[Title/Abstract] OR (Lifestyle[Title/Abstract] OR Lifestyles[Title/Abstract] OR Life Style Induced Illness[Title/Abstract] OR Lifestyle Factors[Title/Abstract] OR Factor, Lifestyle[Title/Abstract] OR Lifestyle Factor[Title/Abstract] OR Lifestyle, Healthy[Title/Abstract] OR Lifestyles, Healthy[Title/Abstract] OR Healthy Life Styles[Title/Abstract] OR Healthy Lifestyles[Title/Abstract] OR Healthy Life Style[Title/Abstract] OR Life Style, Healthy[Title/Abstract] OR Life Styles, Healthy[Title/Abstract] OR Behavior, Sedentary[Title/Abstract] OR Sedentary Behaviors[Title/Abstract] OR Sedentary Lifestyle[Title/Abstract] OR Lifestyle, Sedentary[Title/Abstract] OR Physical Inactivity[Title/Abstract] OR Inactivity, Physical[Title/Abstract] OR Lack of Physical Activity[Title/Abstract] OR Sedentary Time[Title/Abstract] OR Sedentary Times[Title/Abstract] OR Time, Sedentary[Title/Abstract] OR Exercise[Mesh] OR Exercises[Title/Abstract] OR Physical Activity[Title/Abstract] OR Activities, Physical[Title/Abstract] OR Activity, Physical[Title/Abstract] OR Physical Activities[Title/Abstract] OR Exercise, Physical[Title/Abstract] OR Exercises, Physical[Title/Abstract] OR Physical Exercise[Title/Abstract] OR Physical Exercises[Title/Abstract] OR Acute Exercise[Title/Abstract] OR Acute Exercises[Title/Abstract] OR Exercise, Acute[Title/Abstract] OR Exercises, Acute[Title/Abstract] OR Exercise, Isometric[Title/Abstract] OR Exercises, Isometric[Title/Abstract] OR Isometric Exercises[Title/Abstract] OR Isometric Exercise[Title/Abstract] OR Exercise, Aerobic[Title/Abstract] OR Aerobic Exercise[Title/Abstract] OR Aerobic Exercises[Title/Abstract] OR Exercises, Aerobic[Title/Abstract] OR Exercise Training[Title/Abstract] OR Exercise Trainings[Title/Abstract] OR Training, Exercise[Title/Abstract] OR Trainings, Exercise[Title/Abstract] OR Diet[Mesh] OR diets[Title/Abstract] OR Sleep[Mesh] OR Sleeping Habits[Title/Abstract] OR Sleep Habits[Title/Abstract] OR Habit, Sleep[Title/Abstract] OR Habits, Sleep[Title/Abstract] OR Sleep Habit[Title/Abstract] OR Sleeping Habit[Title/Abstract] OR (Habit, Sleeping[Title/Abstract] OR (Habits, Sleeping[Title/Abstract]	887252
3. RCT	randomized controlled trial [Publication Type] OR randomized [Title/Abstract] OR placebo[Title/Abstract]	888684
#1 AND #2 AND #3		503

### 2.3 Assessment of methodological quality

**Figure 2:**
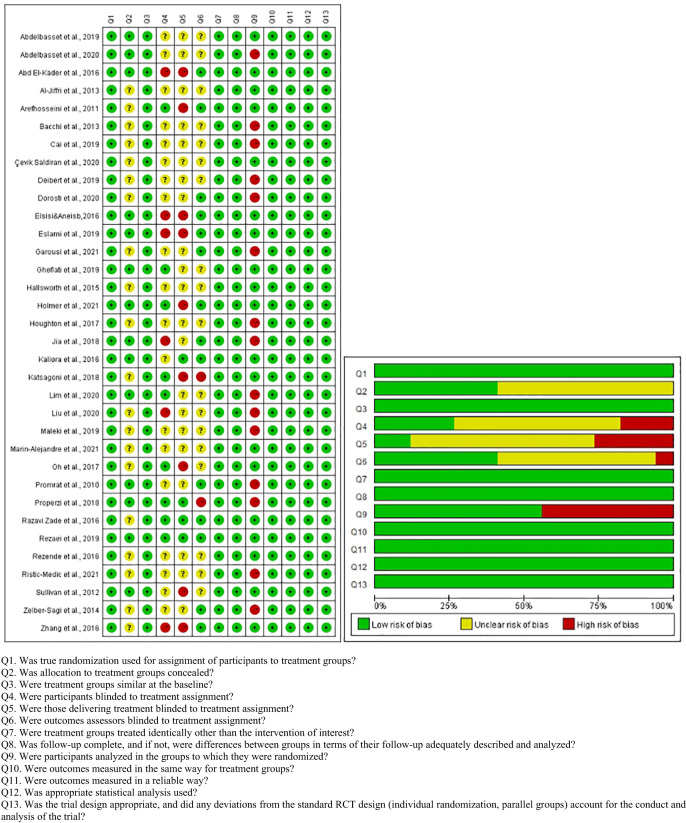
Methodological quality of included studies.

The quality of the included articles was assessed by two independent researchers using the Joanna Briggs Institute Critical Appraisal tools for Checklist for RCTs, which contain 13 entries (**Figure 2**). Each entry was answered with yes, no, or unclear.

### 2.4 Data extraction

All data were extracted by two independent researchers by using standardized forms, and disagreements were resolved by a third researcher. The standard form included authors and year of publication, country, study design, subject, diagnosis methods, sample size, the intervention of experimental and control groups, duration, outcomes, results, and conclusion. Among the articles included in the systematic review, those studies that provided intervention values of HFC, BMI, WC, BP, TG, HDL, FG, HbA1c, and HOMA-IR were included in the meta-analysis.

### 2.5 Data synthesis

ReviewManager 5.3 version was used for data consolidation, heterogeneity test, sensitivity analysis, forest map mapping, and subgroup analysis. First, clinical heterogeneity was determined, and if heterogeneity existed, meta-analysis was discarded, and the results were presented in the form of text and detailed tabular summaries. Secondly, statistical heterogeneity could be explored by chi-square test after excluding clinical heterogeneity. The results were presented as standardized mean difference (SMD) with 95% confidence intervals (CIs), and P<0.05 was considered statistically significant. A fixed-effects model was applied when the heterogeneity test indicated no significant difference (P>0.1 and I^2^<50%); otherwise, a random-effects model was used. Meta-analysis was abandoned, and descriptive methods were used when the source of heterogeneity could not be determined.

## 3 Results

### 3.1 Study selection and study characteristics

The PRISMA flow diagram summarized the selection process of the systematic review and meta-analysis (as shown in **Figure 1**). A total of 8469 studies were identified through search in nine databases. Duplicate references were identified and removed (N=3620). After examining the titles and abstracts, 252 full texts were further screened, and 34 studies met the inclusion criteria and were included in the systematic review and meta-analysis. The reasons for exclusion at this full-text level (N=218) are listed in **Figure 1**.

The characteristics of the included studies are shown in **Table 2**. The 34 included studies reported 2929 adults with MAFLD, and 27 of them reported information about participants lost at follow-up; the final total number was 2652. For diagnosis of MAFLD, 93% of them were based on imaging-detected liver fat accumulation combined with obesity; the remaining 7% were biopsy-proven liver fat accumulation combined with obesity. Moreover, all participants were obesity, and 8% of them had a diagnosis of type 2 diabetes. The sample size of the participants ranged from 18 to 461, and the age of participants ranged from 27 to 68 years. We classified these articles into three categories on the basis of the form of lifestyle interventions: diet interventions, exercise interventions, and diet combined with exercise interventions, which are detailed in **Tables 3**–**5**, respectively.

**Table 2:** Characteristics of the included articles.

Reference		Clinical group	Country	N	Age		M/F	BMI	Intervention form
(25)		Obesity and biopsy-proven NASH	USA	31/28	35–60		22/9	34(25~40)	Diet combined with exercise
(26)		Obesity and ultrasonography-proven NAFLD	Iran	44	30–49		22/22	28.99 ± 2.72/29.21 ± 2.61	Diet
(9)		Obesity and MRI-proven NAFLD	USA	33/18	44–50		5/13	38.1 ± 4.6	Exercise
(27)		Obesity, DM2, and biopsy-proven steatosis	Saudi Arabia	100	35–55		100/0	32.11 ± 3.54/32.37 ± 3.92	Diet combined with exercise
(28)		Obesity, DM2, and MRI scan-proven NAFLD	Italy	32/31	53–58		22/9	30.5 ± 1.0/28.8 ± 1.1	Exercise
(10)		Obesity and ultrasonography-proven NAFLD	Israel	82/64	36–58		34/30	30.75 ± 4.52/31.3 ± 4.14	Exercise
(29)		Obesity and fibrosis scan-proven NAFLD	UK	29/23	44–64		U	31 ± 4/31 ± 5	Exercise
(30)		Obesity and ultrasonography-proven NAFLD	Egypt	32	33–56		15/17	33.99 ± 2.57/33.13 ± 2.11	Exercise
(16)		Obesity and ultrasonography-proven NAFLD	Greece	55/44	40–61		23/32	29.5 ± 4.3/28.2 ± 3.7	Diet
(11)		Obesity and ultrasonography-proven NAFLD	Iran	60/60	32–53		30/30	28.5 ± 3.2/28.3 ± 3.3	Diet
(12)		Obesity and ultrasonography-proven NAFLD	China	220/211/208	46–62		71/149	27.9 ± 2.7/28.1 ± 3.3/28.0 ± 2.7	Exercise
(31)		Obesity and ultrasonography-proven NAFLD	Saudi Arabia	100	45–57		70/30	32.35 ± 2.54/31.76 ± 2.92	Diet combined with exercise
(32)		Obesity and fibrosis scan-proven NAFLD	Brazil	44/40	48–63		0/40	34.1 ± 4.4/32.0 ± 5.0	Exercise
(33)		Obesity and biopsy-proven NASH	Australia	26/24	42–67		U	33 ± 7/33 ± 5	Exercise
(34)		Obesity and fibrosis scan-proven NAFLD	Japan	61/52	46–53		52/0	27.2 ± 0.9/28.4 ± 0.9/28.8 ± 1.1	Exercise
(13)		Obesity and ultrasonography-proven NAFLD	Greece	63/63	38–60		43/20	30.04/31.67/32.44	Diet combined with exercise
(35)		Obesity and ultrasonography-proven NAFLD	China	474/461	48–62		230/231	75.28 ± 10.18/74.94 ± 10.37/74.69 ± 10.3	Exercise
(14)		Obesity and MRI scan-proven NAFLD	Australia	51/50/50/49/48	38–62		25/26	30.2 ± 5.6/31.5 ± 4.1	Diet
(36)		Obesity and ultrasonography-proven NAFLD	Iran	70/64	36–56		19/45	30.90 ± 3.62/31.36 ± 3.74	Diet
(37)		Obesity and ultrasonography-proven NAFLD	Iran	66/54(66)	32–60		29/37	30.6 ± 3.6/29.6 ± 3.9	Diet
(38)		Obesity and ultrasonography-proven NAFLD	Iran	66/62	36–56		19/43	30.85 ± 3.53/31.29 ± 3.77	Diet
(39)		Obesity and ultrasonography-proven NAFLD	Iran	60/54	31–49		12/48	32.77 ± 3.63/31.09 ± 3.24	Diet
(40)		Obesity and MRI-proven NASH	Germany	26/22	40–68		13/9	32.2 ± 3.4/32.4 ± 3.7	Diet combined with exercise
(17)		Obesity and ultrasonography-proven NAFLD	China	271/264	27–41		87/184	26.76 ± 1.59/26.12 ± 2.21/26.34 ± 2.73	Diet
(41)		Obesity, DM2, and MRI scan-proven NAFLD	Saudi Arabia	32/32	49–61		19/13	36.3 ± 4.5/35.9 ± 5.3	Exercise
(15)		Obesity and ultrasonography-proven NAFLD	Iran	112/94	34–52		39/55	32.5 ± 4.1/31.8 ± 4.4	Diet
(18)		Obesity, DM2, and MRI scan-proven NAFLD	Saudi Arabia	48/47	46–63		27/20	36.3 ± 4.5/36.7 ± 3.4/35.9 ± 5.3	Exercise
(42)		Obesity and ultrasonography-proven NAFLD	Singapore	108/101(108)	35–57		68/40	30.1 ± 4.0/30.8 ± 4.8	Diet combined with exercise
(43)		Obesity and fibrosis scan-proven NAFLD	China	158/147	30–55		113/34	27.63 ± 3.46/28.39 ± 2.69/27.11 ± 2.73	Exercise
(44)		Obesity and ultrasonography-proven NAFLD	Turkey	32/31	36–54		12/19	32.74 ± 4.78/33.17 ± 4.91	Exercise
(45)		Obesity and ultrasonography-proven NAFLD	Serbia	27/24	28–39		U	30.43 ± 1.81/30.17 ± 2.28	Diet
(46)		Obesity and imaging-proven NAFLD	Sweden	74/64	47–67		33/41	32.1 ± 3.8/32.3 ± 2.7/32.9 ± 5.2	Diet
(47)		Obesity and ultrasonography-proven NAFLD	Iran	80/75	33–54		36/39	32.02 ± 4.57/30.06 ± 3.81	Diet
(48)		Obesity and ultrasonography-proven NAFLD	Spain	98/76/72/58	41–60		55/43	33.7 ± 4/33.3 ± 4	Diet

The subjects were included in studies and analysis. M/F, male/female; BMI, body mass index; NAFLD, non-alcoholic fatty liver disease; DM2, type-2 diabetes mellitus; MRI, magnetic resonance imaging; CT, computerized tomography; NASH, non-alcoholic steatohepatitis.

**Table 3:** Diet interventions.

Reference	Interventions	Interveners	Control	Duration	Outcomes	Measurer	Results	
(26)	Low carbohydrate diet: carbohydrate:fat:protein: 40:40:20 500 kcal less than energy requirement	Dietitians	Low fat diets: carbohydrate:fat:protein: 55:25:20 500 kcal less than energy requirement	6 weeks	HFC, BMI, TG, HDL	Same trained assessor	TG decreased 18.09% in low carbohydrate diet. HFC and BMI decreased in both groups. Low carbohydrate diet. is better than low fat diet in improving HFC	
(14)	Low carbohydrate diet: carbohydrate:fat:protein:40:40:20	Dietitians	Low-fat diets: carbohydrate:fat:protein: 50:30:20	12 weeks	HFC, BMI, WC, SBP, DBP, TG, HDL, FBG, HbA1c, HOMA-IR	Unmentioned	TG and HbA1c decreased in low carbohydrate diet. HFC decreased in both groups. Low carbohydrate diet. is better than low fat diet in improving HFC	
(45)	Low carbohydrate diet: carbohydrate:fat:protein: 50:30+:15, 600–800 kcal less than energy requirement	Nutritionists	Low-fat diets: carbohydrate:fat:protein: 60:25:15 600–800 kcal less than energy requirement	3 months	HFC, BMI, WC, TG, HDL, FBG, HOMA-IR, CRP	Unmentioned	HFC and TG decreased in low carbohydrate diet. HDL increased in low carbohydrate diet. WC, FBG, and HOMA-IR decreased in both groups.
(46)	Low carbohydrate diet: carbohydrate:fat:protein: 5–10:50–80:10–40	Dietitians	Low-fat diets: carbohydrate:fat:protein: 45–60:20:10–20	12 weeks	HFC, BMI, SBP, DBP, TG, HDL, HbA1c, HOMA-IR	Unmentioned	HFC decreased 7.2% in LCHF diet. HFC decreased 6.1% in 5:2 diet.
(48)	Low carbohydrate diet: carbohydrate:fat:protein: 40–45:30–35:25, 7 meals/day, 30% less than energy requirement	Unmentioned	Low-fat diets: carbohydrate:fat:protein: 55:30:15, 3–5 meals/day, 30% less than energy requirement	24 months	HFC, BMI, WC, SBP, DBP, TG, HDL, HbA1c, HOMA-IR	Unmentioned	HFC, WC, and HOMA-IR decreased in FLiO group.
(16)	Currant replacing snacks of similar caloric content and dietary counseling	Dietitians	Dietary counseling	24 weeks	HFC, BMI, WC, SBP, DBP, TG, HDL, HbA1c	Unmentioned	WC and FBG reduced in the currant arm. HFC, BMI, HbA1c, and CRP reduced in both arms.	
(11)	Rich in fruits, vegetables, whole grains, and low-fat dairy products and low in saturated fats, cholesterol, refined grains, and sweets 350–700 kcal less than energy requirement	Dietitians	Usual diets 350–700 kcal less than energy requirement	8 weeks	HFC, BMI, WC, TG, HDL, FBG, HOMA-IR, CRP	Same trained examiner	BMI, WC, HOMA-IR, TG, and CRP decreased in DASH diet.	
(37)	Olive oil 20 g per day 500 kcal less than energy requirement	Dietitians	Sunflower oil 20 g per day 500 kcal less than energy requirement	12 weeks	HFC, BMI, WC, SBP, DBP, TG, HDL, FBG, HOMA-IR	Unmentioned	HFC and TG reduced in olive group. WC, SBP, and DBP reduced in both groups.	
(36)	Soy milk instead of 1 serving of grains and fats 500 kcal less than energy requirement	Unmentioned	Usual diets 500 kcal less than energy requirement	8 weeks	HFC, BMI, WC, TG, HDL, CRP	Unmentioned	CRP decreased in soy milk group.	
(38)	Soy milk instead of 1 serving of grains and fats 500 kcal less than energy requirement	Unmentioned	Usual diets 500 kcal less than energy requirement	8 weeks	SBP, DBP, FBG, HOMA-IR	Unmentioned	DBP and HOMA-IR reduced in soy milk group.	
(39)	Purslane seeds 10 g/day before breakfast and dinner 500 kcal less than energy requirement	Unmentioned	Usual diets 500 kcal less than energy requirement	8 weeks	SBP, DBP, TG, HDL, FBG, HOMA-IR	Unmentioned	FBG and HOMA-IR reduced in purslane seed group	
(15)	At least half of cereal servings from whole-grain foods each day	Dietitians	Usual cereals	12 weeks	HFC, SBP, DBP, TG, HDL, FBG, HOMA-IR	Same trained assessor	HFC, SBP, and DBP decreased in whole-grain foods	
(17)	A fast day with 25% of energy requirement and a feed day with ad libitum food	Unmentioned	Control: 80% of energy requirement	12 weeks	HFC, BMI, WC, TG, HDL, FBG	Unmentioned	BMI and TG decreased in both ADF and TRF groups.	
	An 8-h window for food intake and a 16-h window of fasting							
(47)	Protein restraining in meat products, 500 kcal less than energy requirement	Unmentioned	Protein freely 500 kcal less than energy requirement	3 months	HFC, BMI, WC, SBP, DBP, TG, HDL, FBG, HOMA-IR	Same trained assessor	HFC, BMI, WC, SBP, TG, FBG, and HOMA-IR decreased in LOV-D.

HFC, hepatic fat content; BMI, body mass index; WC, waist circumference; SBP, systolic blood pressure; DBP, diastolic blood pressure; TG, triglycerides; HDL, high density lipoprotein; FBG, fasting blood glucose; HbA1c, glycated hemoglobin; HOMA-IR, homoeostasis model of assessment-estimated insulin resistance; CRP, high-sensitivity C-reactive protein.

### 3.2 Methodological quality

The quality assessment details of each study are presented in **Figure 2**. Twenty studies did not specify whether allocation concealment was performed, which may cause selection bias. Fifteen studies did not perform intention to treat analysis, potentially leading to follow-up bias. Participants, interveners, and outcome assessors were not fully blinded, probably causing performance bias.

### 3.3 Effects of interventions

#### 3.3.1 Effects of diet interventions

The diet interventions were divided into two categories in this article, as shown in **Table 3**: 1) the first five studies were reduction in the proportion of carbohydrates, and 2) the last nine studies were food serving replacement under isocaloric conditions. A meta-analysis of the diet interventions was deemed inappropriate due to the heterogeneity of the diet forms. We found that low carbohydrate diet significantly improved the level of HFC, TG, HDL, HbA1c, and HOMA-IR, but not BMI, BP, FBG, and CRP when compared to low fat diet. In addition, food serving replacement under isocaloric conditions was most significant in improving HOMA-IR in MAFLD, followed by HFC, WC, TG, FBG, BMI, BP, and CRP, but not HDL and HbA1c.

#### 3.3.2 Effects of exercise interventions

**Figure 3:**
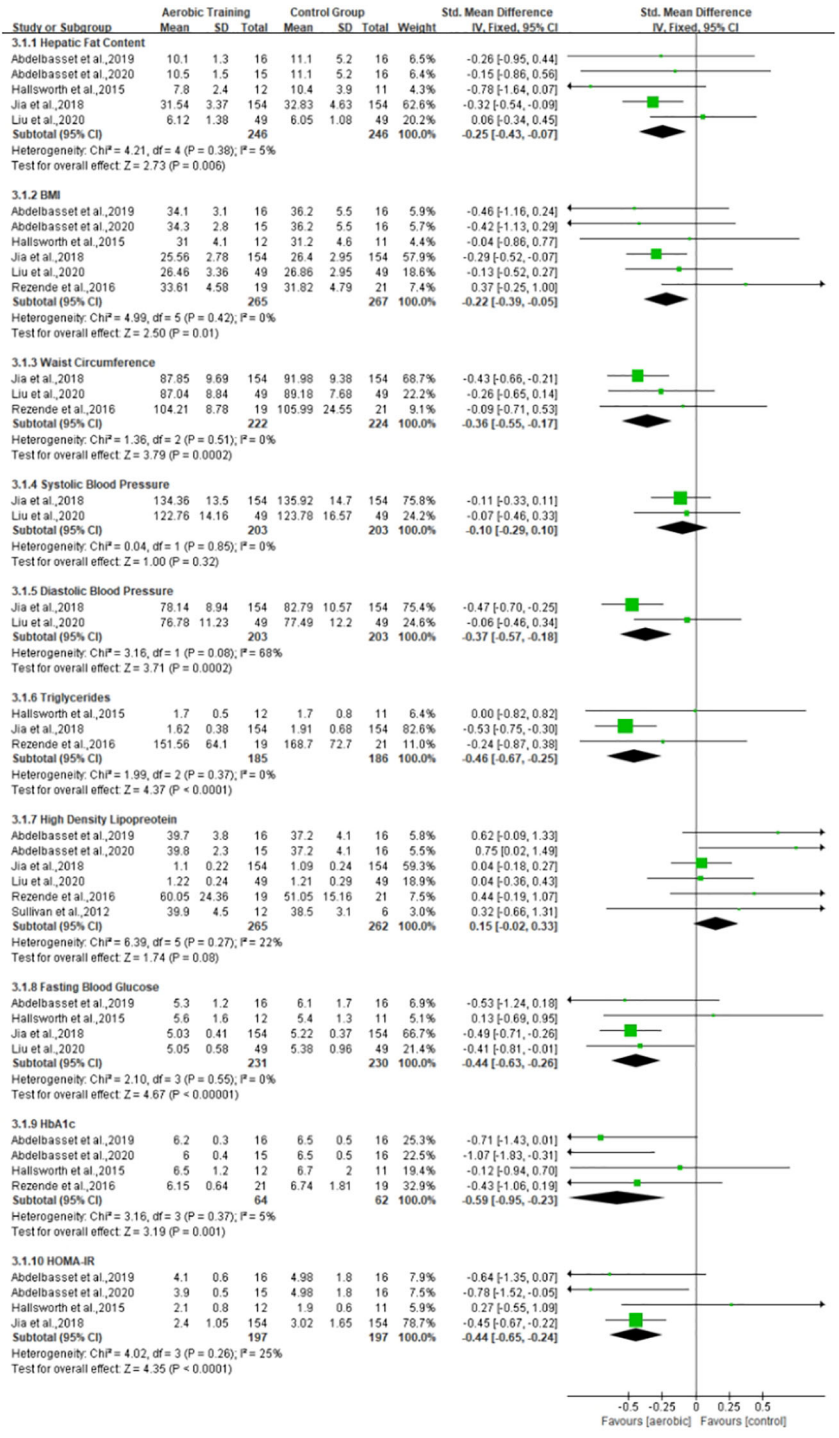
Effect of aerobic training *vs*. control group on MAFLD.

**Figure 4:**
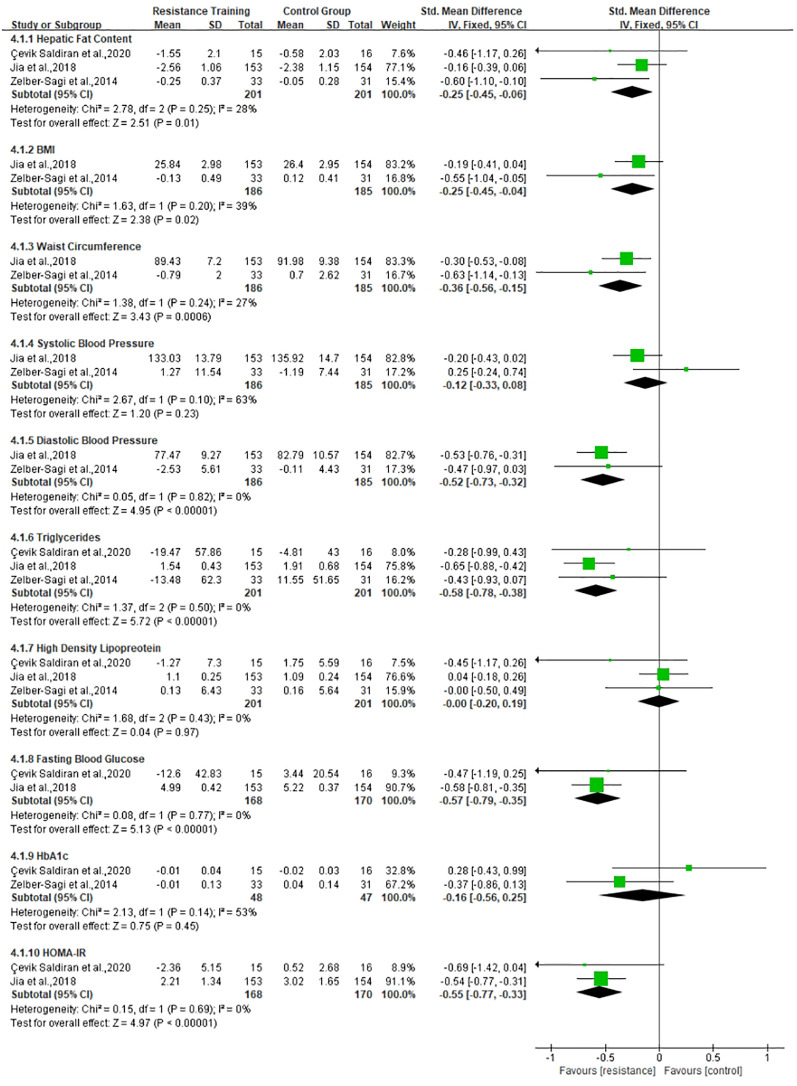
Effect of resistance training *vs*. control group on MAFLD.

**Figure 5:**
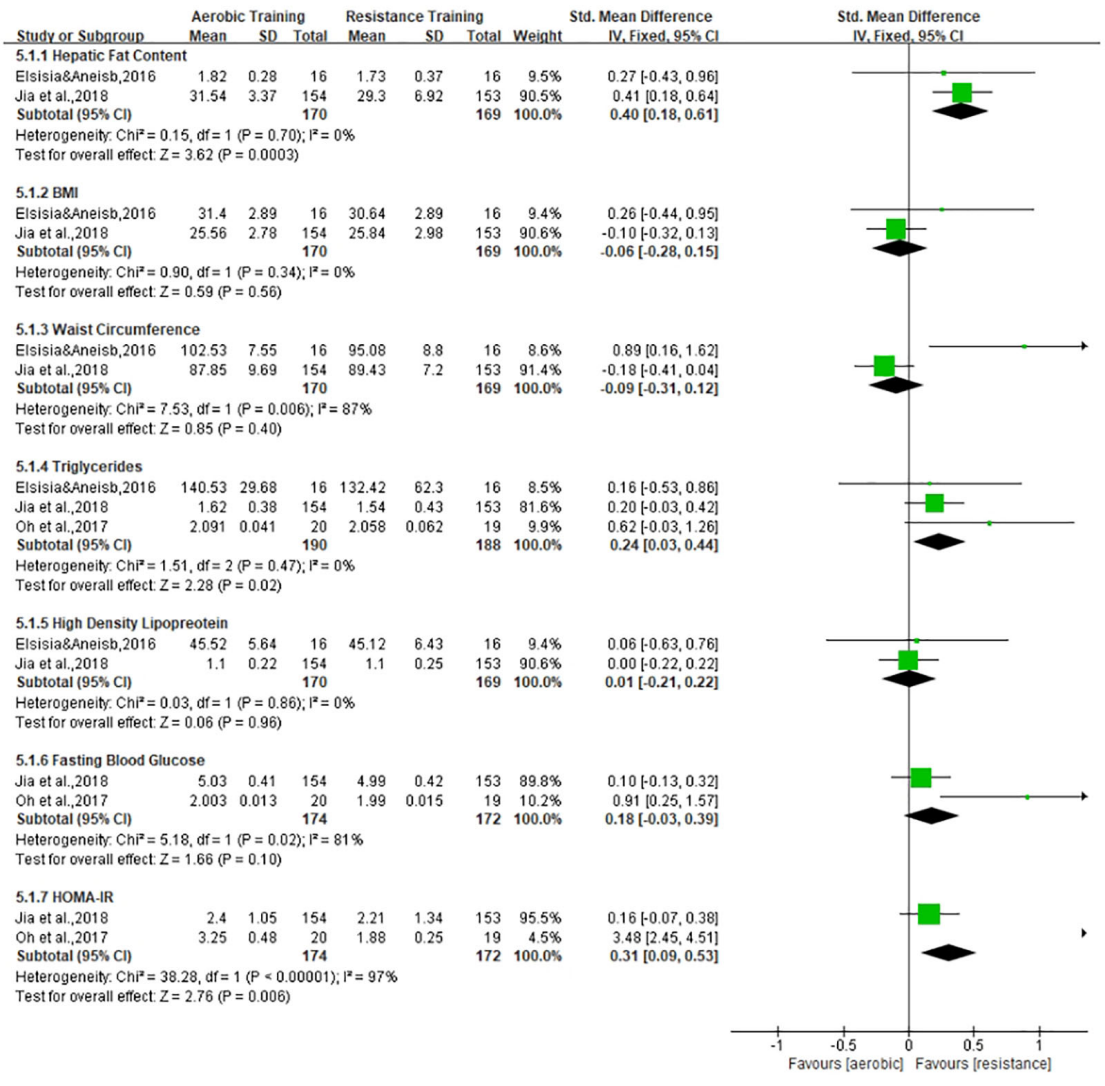
Effect of aerobic training *vs*. resistance training on MAFLD.

The exercise interventions were divided into aerobic training and resistance training, as shown in **Table 4**. According to the different types of exercise, we will conduct meta-analysis in three subgroups: 1) the effects of aerobic training on MAFLD, 2) the effects of resistance exercise on MAFLD, and 3) the difference between aerobic and resistance exercise, which are shown in **Figures 3**–**5**, respectively. Aerobic training was associated with the improvement of the indicator of MAFLD including HFC, BMI, WC, TG, FBG, HbA1c, and HOMA-IR (SMD, -0.25, 95% CI, -0.43 to -0.07; SMD, -0.22, 95% CI, -0.39 to -0.05; SMD, -0.36, 95% CI, -0.55 to -0.17; SMD, -0.46, 95% CI, -0.67 to -0.25; SMD, -0.44, 95% CI, -0.63 to -0.26; SMD, -0.59, 95% CI, -0.95 to -0.23; SMD, -0.44, 95% CI, -0.65 to -0.24, respectively). Resistance training was associated with the improvement of the indicator of MAFLD including HFC, BMI, WC, DBP, TG, FBG, and HOMA-IR (SMD, -0.25, 95% CI, -0.45 to -0.06; SMD, -0.25, 95% CI, -0.56 to -0.15; SMD, -0.36, 95% CI, -0.56 to -0.15; SMD, -0.52, 95% CI, -0.73 to -0.32; SMD, -0.58, 95% CI, -0.78 to -0.38; SMD, -0.57, 95% CI, -0.79 to -0.35; SMD, -0.55, 95% CI, -0.77 to -0.33, respectively). Resistance training was better than aerobic training in reduction in HFC and TG (SMD, -0.25, 95% CI, -0.45 to -0.06; SMD, 0.24, 95% CI, 0.03 to 0.44, respectively). Aerobic and resistance training had no effect on HDL and BP.

**Table 4:** Exercise interventions.

Reference	Types	Intervention			Control		Duration	Outcome	Result
		Types	Intensity	Supervision	Types	Intensity			
(9)	Aerobic training	Aerobic training: 30–60 min×5 times/week	45%–55% of VO2 peak	Under supervision: 1 time/week	Daily activities	Unmentioned	16 weeks	HFC, BMI, TG, HDL	HFC decreased in aerobic training group.
(28)	Both	Aerobic training: 60 min×3 times/week	45%–55% of VO2 peak	Under supervision All	Resistance training: 10repetitions×3×3times/week	70%–80% 1RM	4 months	HFC, BMI, TG, HDL, HbA1c	HFC and HbA1c decreased in both groups.
(10)	Resistance training	Resistance training: 8–12 repetitions×3×3 times/week	Unmentioned	Under supervision ≥2 times	Daily activities	Unmentioned	3 months	HFC, BMI, WC, BP, TG, HDL, FBG, HbA1c	HFC, BMI, and WC decreased in resistance training group.
(29)	Aerobic training	Aerobic training: 30–40min×3 times/week	Unmentioned	Under supervision first 2 sessions	Daily activities	Unmentioned	12 weeks	HFC, BMI, TG, FBG, HbA1c, HOMA-IR	HFC and BMI decreased in HIIT group.
(30)	Both	Resistance exercise: 10 repetitions×2–3×3 times/week	70%–80% 1RM, RPE: 13–14	Under supervision Closely	Aerobic training: 20–30 min×3times/week	75% of max HR	12 weeks	HFC, BMI, WC, TG, HDL	HFC and WC decreased in resistance training group.
(12)	Aerobic training	Aerobic training I: Moderate 6month; 30 min×5 times/week and vigorous 6months 30 min×5times/week×6months	60%–80% of max HR for vigorous exercise; 45%–55% of max HR for moderate exercise	Under supervision 1 time/week	Daily activities	Unmentioned	12 months	HFC, BMI, WC, BP, TG, HDL	WC and SBP reduced in aerobic training I group. HFC reduced in both aerobic training groups.
		Aerobic training II: 30min×5times/week×12months	45%–55% of max HR						
(32)	Aerobic training	Aerobic training: 30–50 min×2times/week	10% below RCP	Under supervision	Daily activities	Unmentioned	24 weeks	HFC, BMI, WC, TG, HDL,HbA1c,HOMA-IR	WC decreased and HDL increased in aerobic group.
(34)	Both	Aerobic trainingI: 3min×3vigorous +2 min×2 rests)×3 times/week	80%–85% of VO2 peak	Unmentioned	Resistance training: 3times/week	1RM	12 weeks	HFC, TG, HOMA-IR	HFC reduced in both aerobic groups.
		Aerobic training II: 40min×3times/week	60%–65% of VO2 peak						
(33)	Both	Aerobic training and/or Resistance training:45–60 min×3 times/week	Aerobic training: RPE: 16–18; Resistance training: RPE: 14–16	Under supervision	Daily activities	Unmentioned	12 weeks	HFC, TG, HbA1c, HOMA-IR, CRP	HFC and TG reduced in interventions group.
(35)	Both	Aerobic training: 45 min×5 times/week,	50%–70% of max HR	Unmentioned	Daily activities	Unmentioned	6 months	HFC, BMI, WC, BP, TG,HLD,FBG,HOMA-IR	HFC, WC, DBP, TG, FBG, and HOMA-IR reduced in both interventions groups.
		Resistance training:: 10–15 repetitions×3×3/week	Unmentioned						
(41)	Aerobic training	Aerobic training: 4 min×3vigorous+2 min ×2 rests)×3/week	80%–85% of VO2 peak	Unmentioned	Daily activities	Unmentioned	8 weeks	HFC, BMI, TG, FBG, HbA1c, HOMA-IR	HFC, FBG, and HbA1c decreased in the aerobic group.
(44)	Resistance training:	Resistance training:: 40 min×4 times/week	60%–80% of max HR	Under supervision	Usual activities	Unmentioned	8 weeks	HFC, TG, HDL, FBG, HbA1c, HOMA-IR	HOMA-IR decreased in resistance training group.
(43)	Aerobic training	Aerobic training 30–60 min×5 times/week	60%–80% of max HR	Under supervision 1 time/week	Daily activities	Unmentioned	3 months	HFC, BMI, WC, BP, TG, FBG	HFC, WC, and TG reduced in aerobic training group.
(18)	Both	Aerobic training I:(4 min×3 vigorous+2 min×2rests)×3times/week	80%–85% of VO2 peak	Under supervision	Daily activities	Unmentioned	8 weeks	HFC, BMI, TG, HDL, HbA1c, HOMA-IR	HFC reduced in both intervention groups.
		Aerobic training II:(4 min×3 ME at +2 min×2rests)×3 times/week	60%–70% of max HR						

HFC, hepatic fat content; BMI, body mass index; WC, waist circumference; SBP, systolic blood pressure; DBP, diastolic blood pressure; TG, triglycerides; HDL, high-density lipoprotein; FBG, fasting blood glucose; HbA1c, glycated hemoglobin; HOMA-IR, homoeostasis model of assessment-estimated insulin resistance; CRP, high-sensitivity C-reactive protein; RPE, rating of perceived exertion; CWT, circuit weight training; HR, heart rate; RCP, respiratory compensation point.

#### 3.3.3 Effects of diet combined with exercise interventions

A meta-analysis of diet combined with exercise interventions was deemed inappropriate due to the heterogeneity of the intervention forms. As shown in **Table 5**, diet combined with exercise interventions was most significant in improving HFC, followed by BMI, HOMA-IR, WC, BP, TG, and HDL. However, diet combined with exercise interventions was not significant in improving FBG and HbA1c.

**Table 5:** Diet combined with exercise interventions.

Reference	Intervention	Control	Duration	Outcome Measures	Result
(25)	Diet: 1000–1500 kcal/day, 25% from fat Exercise: 10,000 steps/day, 200 min/week	Usual lifestyle	48 weeks	HFC, BMI, WC, TG, HDL, HbA1c, HOMA-IR	HFC reduced in intervention group.
(27)	Diet: 1200 kcal/day, Carbohydrate:fat:protein: 55:30–35:15 Exercise: 40 min×36 times, 65%–75% of maximum HR	No intervention	3 months	BMI, HOMA-IR	BMI and HOMA-IR reduced in intervention group.
(31)	Diet: 1200 kcal/day, carbohydrate:fat:protein: 55:30–35:15 Exercise: 40 min×3 times/week, 65%–75% of maximum HR	No intervention	3 months	BMI, TG, HDL, HOMA-IR	BMI, TG, and HOMA-IR reduced in intervention group. HDL increased in intervention group.
(13)	Diet: 1500 kcal/day for women, 1800 kcal/day for men, carbohydrate:fat:protein: 45:35:20, Exercise: 30min/day, optimal sleep	Usual lifestyle	6 months	HFC, BMI, TG, HDL, FBG, HOMA-IR	HFC decreased in both intervention groups.
	Diet: 1500 kcal/day for women, 1800 kcal/day for men, carbohydrate:fat:protein: 45:35:20				
(40)	Diet: first 6 weeks: 2 daily meals replace soy–yogurt–honey Following 18 weeks, 1 daily meal replace soy–yogurt–honey	Usual lifestyle	24 weeks	HFC, BMI, WC, HG, HDL, FBG, HbA1c	HFC reduced in intervention group.
(42)	Individualized caloric goal 10,000 steps/day	Usual lifestyle	6 months	BMI, WC, SBP, DBP	WC, SBP, and DBP reduced in intervention group.

HFC, hepatic fat content; BMI, body mass index; WC, waist circumference; SBP, systolic blood pressure; DBP, diastolic blood pressure; TG, triglycerides; HDL, high-density lipoprotein; FBG, fasting blood glucose; HbA1c, glycated hemoglobin; HOMA-IR, homoeostasis model of assessment-estimated insulin resistance.

## 4 Discussion

To our knowledge, this is the first systematic review and meta-analysis examining the effects of lifestyle interventions on adults with MAFLD after the name NAFLD changed to MAFLD and the diagnostic criteria redefined. We find that the intervention effects of low carbohydrate diet are significant on MAFLD. After comparing the effects of aerobic exercise and resistance exercise, resistance exercise is more effective than aerobic exercise in improving HFC and TG on MAFLD in our meta-analysis. We also find diet combined with exercise interventions are significant on MAFLD. However, there are insufficient evidence about the effects of lifestyle intervention on lean/normal weight (49)MAFLD and the effects of lifestyle intervention on certain metabolism-related indicators.

We find the intervention effects of low carbohydrate diet are significant on MAFLD. Our results suggest that low carbohydrate diet is more effective in improving HFC than low fat diet in adults with MAFLD. Although meta-analysis was abandoned for the different proportions of carbohydrates, all the five articles showed significant effects. However, the results of a meta-analysis about the effect of low carbohydrate diet on NAFLD showed no significant difference between low carbohydrate diet group and low-fat diet group (50). The possible reason for the discrepancy would be the changed definition and redefined criteria. The inclusion criteria of participants with MAFLD are based on the new diagnostic criteria, which excludes participants with NAFLD unrelated to metabolic dysfunction, therefore, the participants are more homogeneous. A recent randomized controlled trial on Type 2 Diabetes combined with NAFLD showed that low carbohydrate diet had greater clinically meaningful improvements in glycemic control and weight compared with low fat diet, which is in line with our results. The participants in this RCT meet the diagnostic criteria for MAFLD (49). However, the participants in this RCT are part of the MAFLD, which are not representative of the MAFLD as a whole. Furthermore, the RCT also showed the changes were not sustained 3 months after intervention. The Mechanisms maybe that carbohydrate, the main raw material for liver’s fat synthesis, reduced in diets would inhibit the fat accumulation in hepatocytes, while initiating lipolysis to reduce HFC (51). In a word, it deserves our attention that the results are different after MAFLD redefinition and more researches about comparing low carbohydrate diet with low fat diet are needed.

Our meta-analysis compares the intervention effects of aerobic and resistance training in adults with MAFLD. We find that resistance exercise is more effective than aerobic exercise in improving HFC and TG. This result is supported by a previous meta-analysis (52). Their results showed that resistance exercise leads to more effective outcomes in improving the metabolic syndrome and cardiovascular risk parameters compared to aerobic exercise. Resistance exercise was the most significant effective training method in ameliorating body fat while aerobic exercise was best in improving BMI significantly. The possible reason is that resistance exercise increases muscular strength, endurance, and muscle mass. Muscle mass engaged during exercise stimulates more IL-6 release that has a direct impact on glucose and lipid metabolism (53). However, a recent RCT showed that aerobic training and resistance training with dietary modification are equally effective for reducing HFC and improving underlying insulin resistance among patients with NAFLD (54). The possible reason for the discrepancy would be the different interventions. Hence, future research should distinguish aerobic and resistance exercise and focus on the advantages of resistance exercise in improving HFC and TG.

We also find diet combined with exercise interventions are significant on MAFLD. This finding is consistent with a published systematic review, which shows that a range of lifestyle interventions significantly improve NAFLD (19). Meanwhile, this finding is similar to two other systematic reviews in children with NAFLD, which reports that lifestyle interventions significantly improve NAFLD (22, 23). The results are also in line with a recent RCT study, which shows lifestyle intervention with diet and regular exercise improved functional fitness in middle-aged patients with NAFLD and Metabolic Syndrome (55). However, none studies have compared the differences of diet, exercise and diet combined exercise interventions on MAFLD. Hence, further research should consider diet combined exercise interventions, and comparing their effects from diet only or exercise only intervention on MAFLD.

Our results remind evidence about lean/normal weight MAFLD is insufficient. According to the new diagnostic criteria of MAFLD, MAFLD could be divided into three subgroups, including overweight/obesity, type 2 diabetes and lean/normal weight. We find that all the participants included in this article are obesity, 8% of them have diabetes together, but none is lean or normal weight, which makes the results more applicable to obesity MAFLD rather than lean or normal weight MAFLD. A retrospective cohort study shows improvement in histological liver steatosis on liver biopsy in non-obese patients with NAFLD treated with diet combined with exercise after a mean of 10 weeks (56). A Japanese meta-analysis showed lean NAFLD individuals makeup 20% of the NAFLD population, were older, and had higher mortality (57). A recent meta-analysis also found that a higher mortality in patients with lean NAFLD than those with non-lean NAFLD (58). The results remind that further research about the risk factors and effective interventions of lean NAFLD is warranted. Hence, we suggest that future research should focus not only on obesity MAFLD, but also on lean or normal weight MAFLD.

Our results also remind evidence about certain metabolism-related indicators is needed. NAFLD focus on glutathione aminotransferase, aspartate aminotransferase and fatty fibrosis, while MAFLD focus on HFC and metabolism-related indicators. The outcomes of interest in our study include HFC, WC, BP, TG, HDL, FBG, HbA1c, HOMA-IR, and CRP. However, not all metabolism-related indicators were analyzed in each included study, especially CRP. We could not analysis the effects of lifestyle interventions on CRP in MAFLD according to available data. These suggest that certain metabolism-related indicators deserve attention in future research.

### 4.1 Limitations

This review has several limitations. First, there is moderate heterogeneity among the analyzed studies, in part due to the differences in inclusion criteria and type and duration of lifestyle interventions. Second, not all interesting end points are reported in the included studies. For example, CRP is not reported in a majority of included studies, and the effect of lifestyle on CRP could not be evaluated. Third, selection bias, performance bias, and follow-up bias in some of the analyses could be another limitation. Therefore, the results should be taken with caution, and more studies on the effect of lifestyle interventions are required to reinforce the recommendations of lifestyle in the treatment and prevention of MAFLD.

### 4.2 Implications for practice

The recommendations for future research are provided as follows. First, the MAFLD subjects can be divided into overweight/obesity, type 2 diabetes, or lean/normal weight, and subjects with lean/normal weight MAFLD should be focused more because they are more likely to be overlooked and there is insufficient evidence from existing studies. Second, lifestyle interventions should include both diet and exercise and respect for preferences of MAFLD; for example, resistance training may be more feasible than aerobic training for MAFLD patients who are less fit or unable to tolerate aerobic training. Lastly, future studies should focus more on all metabolism-related indicators, especially CRP.

## 5 Conclusion

This systematic review and meta-analysis examine the effects of lifestyle interventions on adults MAFLD for the first time after the name changed. Low carbohydrate diet is more effective in improving HFC than low fat diet in adults with MAFLD. Resistance exercise is more effective than aerobic exercise in improving HFC and TG in adults MAFLD. Diet combined with exercise interventions are significant on MAFLD. However, our results more applicable to obesity MAFLD rather than lean or normal weight MAFLD.

## Data availability statement

The original contributions presented in the study are included in the article/supplementary material. Further inquiries can be directed to the corresponding author.

## Author contributions

X-NC: Data Curation, Formal analysis, Writing - original draft. B-QZ: Data Curation, Validation, Investigation. NN: Data Curation, Validation. TP: Formal analysis. FX: Formal analysis. S-HH: Formal analysis. N-NC: Formal analysis. MS: Conceptualization; Methodology; Writing - review and editing. All authors contributed to the article and approved the submitted version.

## References

[1] Chalasani N Younossi Z Lavine JE Charlton M Cusi K Rinella M . The diagnosis and management of nonalcoholic fatty liver disease: Practice guidance from the American association for the study of liver diseases. Hepatology (2018) 67(1):328–57. doi: 10.1002/hep.29367 28714183

[2] Eslam M Newsome PN Sarin SK Anstee QM Targher G Romero-Gomez M . A new definition for metabolic dysfunction-associated fatty liver disease: An international expert consensus statement. J Hepatol (2020) 73(1):202–9. doi: 10.1016/j.jhep.2020.03.039 32278004

[3] Eslam M Sarin SK Wong VW Fan JG Kawaguchi T Ahn SH . The Asian pacific association for the study of the liver clinical practice guidelines for the diagnosis and management of metabolic associated fatty liver disease. Hepatol Int (2020) 14(6):889–919. doi: 10.1007/s12072-020-10094-2 33006093

[4] Fazel Y Koenig AB Sayiner M Goodman ZD Younossi ZM . Epidemiology and natural history of non-alcoholic fatty liver disease. Metabolism (2016) 65(8):1017–25. doi: 10.1016/j.metabol.2016.01.012 26997539

[5] Younossi Z Anstee QM Marietti M Hardy T Henry L Eslam M . Global burden of NAFLD and NASH: trends, predictions, risk factors and prevention. Nat Rev Gastroenterol Hepatol (2018) 15(1):11–20. doi: 10.1038/nrgastro.2017.109 28930295

[6] Younossi Z Tacke F Arrese M Chander Sharma B Mostafa I Bugianesi E . Global perspectives on nonalcoholic fatty liver disease and nonalcoholic steatohepatitis. Hepatology (2019) 69(6):2672–82. doi: 10.1002/hep.30251 30179269

[7] Inoue Y Qin B Poti J Sokol R Gordon-Larsen P . Epidemiology of obesity in adults: Latest trends. Curr Obes Rep (2018) 7(4):276–88. doi: 10.1007/s13679-018-0317-8 PMC6215729 30155850

[8] EASL-EASD-EASO . EASL-EASD-EASO clinical practice guidelines for the management of non-alcoholic fatty liver disease. Diabetologia (2016) 59(6):1121–40. doi: 10.1007/s00125-016-3902-y 27053230

[9] Sullivan S Kirk EP Mittendorfer B Patterson BW Klein S . Randomized trial of exercise effect on intrahepatic triglyceride content and lipid kinetics in nonalcoholic fatty liver disease. Hepatology (2012) 55(6):1738–45. doi: 10.1002/hep.25548 PMC3337888 22213436

[10] Zelber-Sagi S Buch A Yeshua H Vaisman N Webb M Harari G . Effect of resistance training on non-alcoholic fatty-liver disease a randomized-clinical trial. World J Gastroenterol (2014) 20(15):4382–92. doi: 10.3748/wjg.v20.i15.4382 PMC3989975 24764677

[11] Razavi Zade M Telkabadi MH Bahmani F Salehi B Farshbaf S Asemi Z . The effects of DASH diet on weight loss and metabolic status in adults with non-alcoholic fatty liver disease: a randomized clinical trial. Liver Int (2016) 36(4):563–71. doi: 10.1111/liv.12990 26503843

[12] Zhang HJ He J Pan LL Ma ZM Han CK Chen CS . Effects of moderate and vigorous exercise on nonalcoholic fatty liver disease: A randomized clinical trial. JAMA Intern Med (2016) 176(8):1074–82. doi: 10.1001/jamainternmed.2016.3202 27379904

[13] Katsagoni CN Papatheodoridis GV Ioannidou P Deutsch M Alexopoulou A Papadopoulos N . Improvements in clinical characteristics of patients with non-alcoholic fatty liver disease, after an intervention based on the Mediterranean lifestyle: a randomised controlled clinical trial. Br J Nutr (2018) 120(2):164–75. doi: 10.1017/S000711451800137X 29947322

[14] Properzi C O’Sullivan TA Sherriff JL Ching HL Jeffrey GP Buckley RF . Ad libitum Mediterranean and low-fat diets both significantly reduce hepatic steatosis: A randomized controlled trial. Hepatology (2018) 68(5):1741–54. doi: 10.1002/hep.30076 29729189

[15] Dorosti M Jafary Heidarloo A Bakhshimoghaddam F Alizadeh M . Whole-grain consumption and its effects on hepatic steatosis and liver enzymes in patients with non-alcoholic fatty liver disease: a randomised controlled clinical trial. Br J Nutr (2020) 123(3):328–36. doi: 10.1017/S0007114519002769 31685037

[16] Kaliora AC Kokkinos A Diolintzi A Stoupaki M Gioxari A Kanellos PT . The effect of minimal dietary changes with raisins in NAFLD patients with non-significant fibrosis: a randomized controlled intervention. Food Funct (2016) 7(11):4533–44. doi: 10.1039/c6fo01040g 27714002

[17] Cai H Qin YL Shi ZY Chen JH Zeng MJ Zhou W . Effects of alternate-day fasting on body weight and dyslipidaemia in patients with non-alcoholic fatty liver disease: a randomised controlled trial. BMC Gastroenterol (2019) 19(1):219. doi: 10.1186/s12876-019-1132-8 31852444 PMC6921505

[18] Abdelbasset WK Tantawy SA Kamel DM Alqahtani BA Elnegamy TE Soliman GS . Effects of high-intensity interval and moderate-intensity continuous aerobic exercise on diabetic obese patients with nonalcoholic fatty liver disease: A comparative randomized controlled trial. Med (Baltimore) (2020) 99(10):e19471. doi: 10.1097/MD.0000000000019471 PMC7478706 32150108

[19] Thoma C Day CP Trenell MI . Lifestyle interventions for the treatment of non-alcoholic fatty liver disease in adults: a systematic review. J Hepatol (2012) 56(1):255–66. doi: 10.1016/j.jhep.2011.06.010 21723839

[20] Orci LA Gariani K Oldani G Delaune V Morel P Toso C . Exercise-based interventions for nonalcoholic fatty liver disease: A meta-analysis and meta-regression. Clin Gastroenterol Hepatol (2016) 14(10):1398–411. doi: 10.1016/j.cgh.2016.04.036 27155553

[21] Medrano M Cadenas-Sanchez C Alvarez-Bueno C Cavero-Redondo I Ruiz JR Ortega FB . Evidence-based exercise recommendations to reduce hepatic fat content in youth- a systematic review and meta-analysis. Prog Cardiovasc Dis (2018) 61(2):222–31. doi: 10.1016/j.pcad.2018.01.013 29452135

[22] Utz-Melere M Targa-Ferreira C Lessa-Horta B Epifanio M Mouzaki M Mattos AA . Non-alcoholic fatty liver disease in children and adolescents: Lifestyle change - a systematic review and meta-analysis. Ann Hepatol (2018) 17(3):345–54. doi: 10.5604/01.3001.0011.7380 29735796

[23] Mann JP Tang GY Nobili V Armstrong MJ . Evaluations of lifestyle, dietary, and pharmacologic treatments for pediatric nonalcoholic fatty liver disease: A systematic review. Clin Gastroenterol Hepatol (2019) 17(8):1457–1476 e1457. doi: 10.1016/j.cgh.2018.05.023 29857146

[24] Liberati A Altman DG Tetzlaff J Mulrow C Gotzsche PC Ioannidis JP . The PRISMA statement for reporting systematic reviews and meta-analyses of studies that evaluate healthcare interventions: explanation and elaboration. BMJ (2009) 339:b2700. doi: 10.1136/bmj.b2700 19622552 PMC2714672

[25] Promrat K Kleiner DE Niemeier HM Jackvony E Kearns M Wands JR . Randomized controlled trial testing the effects of weight loss on nonalcoholic steatohepatitis. Hepatology (2010) 51(1):121–9. doi: 10.1002/hep.23276 PMC2799538 19827166

[26] Arefhosseini SR Ebrahimi-Mameghani M Farsad Naeimi A Khoshbaten M Rashid J . Lifestyle modification through dietary intervention: Health promotion of patients with non-alcoholic fatty liver disease. Health Promot Perspect (2011) 1(2):147–54. doi: 10.5681/hpp.2011.016 PMC3963618 24688911

[27] Al-Jiffri O Al-Sharif FM Abd El-Kader SM Ashmawy EM . Weight reduction improves markers of hepatic function and insulin resistance in type-2 diabetic patients with non-alcoholic fatty liver. Afr Health Sci (2013) 13(3):667–72. doi: 10.4314/ahs.v13i3.21 PMC3824460 24250305

[28] Bacchi E Negri C Targher G Faccioli N Lanza M Zoppini G . Both resistance training and aerobic training reduce hepatic fat content in type 2 diabetic subjects with nonalcoholic fatty liver disease (the RAED2 randomized trial). Hepatology (2013) 58(4):1287–95. doi: 10.1002/hep.26393 23504926

[29] Hallsworth K Thoma C Hollingsworth KG Cassidy S Anstee QM Day CP . Modified high-intensity interval training reduces liver fat and improves cardiac function in non-alcoholic fatty liver disease: a randomized controlled trial. Clin Sci (Lond) (2015) 129(12):1097–105. doi: 10.1042/CS20150308 26265792

[30] Elsisia HF Aneisb YM . High-intensity circuit weight training versus aerobic training in patients with nonalcoholic fatty liver disease. Bull Faculty Phys Ther (2016) 20(2):181–92. doi: 10.4103/1110-6611.174717

[31] Abd El-Kader SM Al-Shreef FM Al-Jiffri OH . Biochemical parameters response to weight loss in patients with non-alcoholic steatohepatitis. Afr Health Sci (2016) 16(1):242–9. doi: 10.4314/ahs.v16i1.32 PMC4915432 27358638

[32] Rezende RE Duarte SM Stefano JT Roschel H Gualano B de Sa Pinto AL . Randomized clinical trial: benefits of aerobic physical activity for 24 weeks in postmenopausal women with nonalcoholic fatty liver disease. Menopause (2016) 23(8):876–83. doi: 10.1097/GME.0000000000000647 27458060

[33] Houghton D Thoma C Hallsworth K Cassidy S Hardy T Burt AD . Exercise reduces liver lipids and visceral adiposity in patients with nonalcoholic steatohepatitis in a randomized controlled trial. Clin Gastroenterol Hepatol (2017) 15(1):96–102.e103. doi: 10.1016/j.cgh.2016.07.031 27521509 PMC5196006

[34] Oh S So R Shida T Matsuo T Kim B Akiyama K . High-intensity aerobic exercise improves both hepatic fat content and stiffness in sedentary obese men with nonalcoholic fatty liver disease. Sci Rep (2017) 7:43029. doi: 10.1038/srep43029 28223710 PMC5320441

[35] Jia GY Han T Gao L Wang L Wang SC Yang L . A randomized controlled study of aerobic and resistance exercise to improve nonalcoholic fatty liver disease. Chin J Liver Dis (2018) 26(01):34–41. doi: 10.3760/cma.j.issn.1007-3418.2018.01.009 PMC12769912 29804360

[36] Eslami O Shidfar F Maleki Z Jazayeri S Hosseini AF Agah S . Effect of soy milk on metabolic status of patients with nonalcoholic fatty liver disease: A randomized clinical trial. J Am Coll Nutr (2019) 38(1):51–8. doi: 10.1080/07315724.2018.1479990 30028245

[37] Rezaei S Akhlaghi M Sasani MR Barati Boldaji R . Olive oil lessened fatty liver severity independent of cardiometabolic correction in patients with non-alcoholic fatty liver disease: A randomized clinical trial. Nutrition (2019) 57:154–61. doi: 10.1016/j.nut.2018.02.021 30170304

[38] Maleki Z Jazayeri S Eslami O Shidfar F Hosseini AF Agah S . Effect of soy milk consumption on glycemic status, blood pressure, fibrinogen and malondialdehyde in patients with non-alcoholic fatty liver disease: a randomized controlled trial. Complement Ther Med (2019) 44:44–50. doi: 10.1016/j.ctim.2019.02.020 31126574

[39] Gheflati A Adelnia E Nadjarzadeh A . The clinical effects of purslane (Portulaca oleracea) seeds on metabolic profiles in patients with nonalcoholic fatty liver disease: A randomized controlled clinical trial. Phytother Res (2019) 33(5):1501–9. doi: 10.1002/ptr.6342 30895694

[40] Deibert P Lazaro A Schaffner D Berg A Koenig D Kreisel W . Comprehensive lifestyle intervention vs soy protein-based meal regimen in non-alcoholic steatohepatitis. World J Gastroenterol (2019) 25(9):1116–31. doi: 10.3748/wjg.v25.i9.1116 PMC6406181 30862999

[41] Abdelbasset WK Tantawy SA Kamel DM Alqahtani BA Soliman GS . A randomized controlled trial on the effectiveness of 8-week high-intensity interval exercise on intrahepatic triglycerides, visceral lipids, and health-related quality of life in diabetic obese patients with nonalcoholic fatty liver disease. Med (Baltimore) (2019) 98(12):e14918. doi: 10.1097/MD.0000000000014918 PMC6708753 30896648

[42] Lim SL Johal J Ong KW Han CY Chan YH Lee YM . Lifestyle intervention enabled by mobile technology on weight loss in patients with nonalcoholic fatty liver disease: Randomized controlled trial. JMIR Mhealth Uhealth (2020) 8(4):e14802. doi: 10.2196/14802 32281943 PMC7186867

[43] Liu YY Liu YP Liu YR Sun P . A prospective study on the implementation of aerobic exercise intervention in patients with metabolism-related fatty liver disease. J Clin Hepatol (2020) 36(11):2467–72. doi: 10.3969/j.issn.1001-5256.2020.11.014

[44] Çevik Saldiran T Mutluay FK Yağci İ. Yilmaz Y . Impact of aerobic training with and without whole-body vibration training on metabolic features and quality of life in non-alcoholic fatty liver disease patients. Ann Endocrinol (Paris) (2020) 81(5):493–9. doi: 10.1016/j.ando.2020.05.003 32768394

[45] Ristic-Medic D Kovacic M Takic M Arsic A Petrovic S Paunovic M . Calorie-restricted Mediterranean and low-fat diets affect fatty acid status in individuals with nonalcoholic fatty liver disease. Nutrients (2020) 13(1):15. doi: 10.3390/nu13010015 33374554 PMC7822481

[46] Holmer M Lindqvist C Petersson S Moshtaghi-Svensson J Tillander V Brismar TB . Treatment of NAFLD with intermittent calorie restriction or low-carb high-fat diet - a randomised controlled trial. JHEP Rep (2021) 3(3):100256. doi: 10.1016/j.jhepr.2021.100256 33898960 PMC8059083

[47] Garousi N Tamizifar B Pourmasoumi M Feizi A Askari G Clark CCT . Effects of lacto-ovo-vegetarian diet vs. standard-weight-loss diet on obese and overweight adults with non-alcoholic fatty liver disease: a randomised clinical trial. Arch Physiol Biochem (2021) 9:1–9. doi: 10.1080/13813455.2021.1890128 33689525

[48] Marin-Alejandre BA Cantero I Perez-Diaz-Del-Campo N Monreal JI Elorz M Herrero JI . Effects of two personalized dietary strategies during a 2-year intervention in subjects with nonalcoholic fatty liver disease: A randomized trial. Liver Int (2021) 41(7):1532–44. doi: 10.1111/liv.14818 33550706

[49] Hansen CD Gram-Kampmann EM Hansen JK Hugger MB Madsen BS Jensen JM . Effect of calorie-unrestricted low-carbohydrate, high-fat diet versus high-carbohydrate, low-fat diet on type 2 diabetes and nonalcoholic fatty liver disease. Ann Internal Med (2023) 176(1):10–22. doi: 10.7326/P22-0022 36508737

[50] Ahn J Jun DW Lee HY Moon JH . Critical appraisal for low-carbohydrate diet in nonalcoholic fatty liver disease: Review and meta-analyses. Clin Nutr (2019) 38(5):2023–30. doi: 10.1016/j.clnu.2018.09.022 30314924

[51] Brouns F . Overweight and diabetes prevention: is a low-carbohydrate-high-fat diet recommendable? Eur J Nutr (2018) 57(4):1301–12. doi: 10.1007/s00394-018-1636-y PMC5959976 29541907

[52] Liang M Pan Y Zhong T Zeng Y Cheng ASK . Effects of aerobic, resistance, and combined exercise on metabolic syndrome parameters and cardiovascular risk factors: a systematic review and network meta-analysis. Rev Cardiovasc Med (2021) 22(4):1523–33. doi: 10.31083/j.rcm2204156 34957791

[53] Pedersen BK . Anti-inflammatory effects of exercise: role in diabetes and cardiovascular disease. Eur J Clin Invest (2017) 47(8):600–11. doi: 10.1111/eci.12781 28722106

[54] Charatcharoenwitthaya P Kuljiratitikal K Aksornchanya O Chaiyasoot K Bandidniyamanon W Charatcharoenwitthaya N . Moderate-intensity aerobic vs resistance exercise and dietary modification in patients with nonalcoholic fatty liver disease: A randomized clinical trial. Clin Trans Gastroenterol (2021) 12:e00316. doi: 10.14309/ctg.0000000000000316 PMC7925136 33939383

[55] Mascaró CM Bouzas C Montemayor S Casares M Llompart I Ugarriza L . Effect of a six-month lifestyle intervention on the physical activity and fitness status of adults with NAFLD and metabolic syndrome. Nutrients (2022) 14(9):1813. doi: 10.3390/nu14091813 35565780 PMC9105030

[56] Jin YJ Kim KM Hwang S Lee SG Ha TY Song GW . Exercise and diet modification in non-obese non-alcoholic fatty liver disease: analysis of biopsies of living liver donors. J Gastroenterol Hepatol (2012) 27(8):1341–7. doi: 10.1111/j.1440-1746.2012.07165.x 22554085

[57] Ito T Ishigami M Zou B Tanaka T Takahashi H Kurosaki M . The epidemiology of NAFLD and lean NAFLD in Japan: a meta-analysis with individual and forecasting analysis 1995-2040. Hepatol Int (2021) 15(2):366–79. doi: 10.1007/s12072-021-10143-4 33580453

[58] Ha J Yim SY Karagozian R . Mortality and liver-related events in lean versus non-lean nonalcoholic fatty liver disease: A systematic review and meta-analysis. Clin Gastroenterol Hepatol (2022) 26:S1542-3565(22)01099-0. doi: 10.1016/j.cgh.2022.11.019 36442727

